# Increasing physician participation as subjects in scientific and quality improvement research

**DOI:** 10.1186/s12910-022-00817-5

**Published:** 2022-08-13

**Authors:** Sylvia J. Hysong, Amy L. McGuire

**Affiliations:** 1 Baylor College of Medicine, 1 Baylor Plaza, Houston, TX 77030 USA; 2 Center for Innovations in Quality, Effectiveness and Safety, Michael E. DeBakey VA Medical Center, 2002 Holcombe Blvd. (152), Houston, TX USA

**Keywords:** Physicians, Research subjects, Incentives, Quality improvement

## Abstract

**Background:**

The twenty-first century has witnessed an exponential increase in healthcare quality research. As such activities become more prevalent, physicians are increasingly needed to participate as subjects in research and quality improvement (QI) projects. This raises an important ethical question: how should physicians be remunerated for participating as research and/or QI subjects?

**Financial versus non-monetary incentives for participation:**

Research suggests participation in research and QI is often driven by conditional altruism, the idea that although initial interest in enrolling in research is altruistic or prosocial, decisions to actually perform study tasks are cost–benefit driven. Thus, the three models commonly employed to appropriately compensate participants (in-kind compensation such as travel reimbursement, paying market rates for the subject’s time, and paying market rates for the activity asked of the participant) are a poor fit when the participant is a clinician, largely due to the asymmetry between cost and benefit or value to the participant. Non-monetary alternatives such as protected time for participation, continuing education or maintenance of certification credit, or professional development materials, can provide viable avenues for reducing this asymmetry.

**Conclusion:**

Research and QI are integral to the betterment of medicine and healthcare. To increase physician participation in these activities as the subject of study, new models are needed that clarify the physician’s role in research and QI as a subject. Non-monetary approaches are recommended to successfully and ethically encourage research and QI participation, and thus incorporate these activities as a normal part of the ethical clinician’s and successful learning healthcare system’s world view.

## Background

The twenty-first century has witnessed marked change in the structure and delivery of healthcare in the western world and a corresponding, exponential increase both in healthcare quality research, defined as systematic investigations intended to contribute to generalizable knowledge, and healthcare quality improvement (QI) work—the systematic collection of quality data and data-driven intervention design intended to directly improve processes and outcomes within a given setting, rather than produce findings applicable to other settings or populations [1]. In PubMed alone, the number of publications returned when the term “healthcare quality improvement” is entered into its search box has grown more than fivefold in the last 20 years, from 209,401 between 1946 and 2002 to 1,089,469 between 2002 and 2022 [2]. As such activities become more prevalent, clinicians are increasingly needed to participate as subjects in research and QI projects, intensifying demand for physicians who, through this act of service, advance the science of long-standing topics such as medical education, professional issues, and (especially in the era of COVID-19), clinician burnout and the well-being of the healthcare workforce. The consequences of under-engagement in either research or QI can be significant; for example, Rawatni and colleagues [3] demonstrated that difficulties enrolling physicians in usability studies have been associated with many of the user interface challenges in modern electronic health records, leading in some cases to significant medical errors. In this era of continuous improvement, diagnostic errors, and healthcare excellence, physician participants are no longer a luxury, but rather a precious resource susceptible to the effects of supply and demand.

Patients and other types of research subjects (e.g., caregivers, consumers) are commonly remunerated for participation to encourage them to enroll in research studies. For example—veterans might receive $50 for a one-hour interview about their experiences as a patient or have all the medical costs of an experimental procedure waived. Yet in the current Declaration of Geneva, physicians pledge to “share medical and scientific knowledge for the benefit of the patient *and the advancement of healthcare*” [4]. This raises an important ethical question: should physicians be remunerated for participating as research and/or QI subjects? The Declaration of Helsinki is silent on issues of compensation, other than requiring transparency of such in human subject research protocols. Other ethics statements provide only slightly more guidance. For example, the American Medical Association (AMA) code of ethics recommends reimbursing subjects for any care or procedures physicians provide form as part of a research study. Similarly, physicians participating as researchers can receive salary remuneration on most grants. However, when serving as subjects, physicians provide neither clinical care nor scientific expertise—they or their organization are the study’s phenomenon of interest, just as when a patient is in the same role. In the role of subjects, clinicians are as vulnerable to coercion, bias, and conflicts of interest as the patients they enroll in their studies, especially if the research is conducted by their employer. Is there a model of compensation that would have the intended effect of motivating clinicians to participate as subjects, without coercion or other unintended ethical consequences?

### Financial incentives for participation as human subjects

Dickert and Grady [5] proposed multiple models to appropriately compensate an individual’s participation in research, including “in-kind” compensation, paying market rates for the participant’s time, and paying market rates for the activity being asked of the participants. These models usually are for tasks of significant burden and assume the research subject is a patient. None of these is a good fit when the subject is a clinician.

Physicians recruited into a study due to their profession are unlikely to require medical care; thus, reimbursing medical costs is inapplicable, though they could be reimbursed any associated travel costs. Paying market rates for study tasks is also an ill-suited compensation model for clinician subjects, as clinician-centered QI and research tasks tend to consist mostly of surveys, focus groups, or incorporating interventions into their daily workflow. Market rates for such tasks are minimal compared to physician salaries—for example, fair payment guidelines for crowdsourcing workers such as those found through Amazon Mechanical Turk recommend paying approximately ten US (United States) Dollars ($) per hour for survey work—thus providing clinicians with no meaningful financial incentive to participate.

Finally, market rates for clinicians’ (especially physicians’) time, such as through hourly rates or relative value units (RVU) [6], present challenges. No procedure code exists for participating as a research subject or for the types of tasks physicians are likely to perform as subjects, as these are not financially reimbursable. Thus, reimbursing based on RVU may prove difficult to estimate. Conversely, hourly rate compensation, though certainly understandable—time physicians spend as participants is time they are not spending generating revenue—could impose an undue burden on the cost of research. For example, according to the American Association of Medical Colleges the median salary for a physician in clinical sciences is $322,800 [7]. Even a modest goal, such as recruiting 100 physicians for 1 h, would add $16,140 to the budget of a research grant. This could set an unsustainable precedent for research studies and disincentivize practices from conducting research or QI activities.

## Exploring non-monetary alternatives

Given the challenges associated with financial remuneration of clinicians for participating as subjects in scientific research and QI work, we believe financial incentives for clinicians as subjects create more problems than they solve, and thus non-monetary alternatives should be explored. Because motivations for participating in research versus QI are sometimes different, incentivizing clinicians may require different approaches for each type of activity. For example, many clinician-centric QI activities form part of the professional development and continuous improvement any good clinician should practice regularly. Just as continuous medical education is expected of the ethical physician, continuous QI is also part of the successful learning healthcare system. Offering protected time for participating in QI as a subject, not just as a project leader, signals that the physician’s organization values their input sufficiently to clear away barriers in order to obtain it. Protected time, of course, comes at the expense of seeing patients—and generating revenue. Organizations must thus weigh the return on investment of said protected time on outcomes such as improved care quality, access to care, patient satisfaction, practice productivity, or clinician satisfaction.

Conversely, research participation is, ethically, a strictly voluntary activity. Motivations for participating in research, however, have both altruistic (prosocial) and self-interest components. For example, McKann and colleagues [8] suggest research participation is driven by conditional altruism: initial interest in enrolling in research is altruistic or prosocial, but decisions to actually perform study tasks depend on perceiving an individual benefit and no material detriment (i.e., value). From this perspective, physicians may be initially altruistically interested in participating from a powerful sense of professionalism or because they understand the importance of contributing, especially if they are themselves scientists. However, the compensation models discussed may constitute insufficient individual value for physicians to participate, as additional work is being asked of them (detriment) for little to no *additional* monetary benefit (RVU reimbursement would simply mean a temporary change in work, with no additional remuneration). Given competing demands on physicians’ time (lack of time is the most common reason clinicians decline to participate as research subjects) [9], value may be best added non-monetarily (e.g., continuing education or maintenance of certification credit, professional development materials, in-kind services that solve a problem, the luxury of slowing down for a catered lunch on an otherwise jam-packed clinic day), which may prove more effective for recruiting physicians. These types of incentives, however, may require more logistical effort from the research team, still cost money to implement (though likely not as much as monetary incentives), and depending on how implemented, may still cost participants time. For example, offering registration to a professional development conference as an incentive still means the participant must schedule and protect time to attend the conference, in addition to participating as a subject. Thus, logistics and unintended consequences of such nonmonetary incentive must be carefully considered.

An example of a healthcare system that offers navigable, ethical pathways to physician participation as subjects in research and QI is the Veterans Health Administration (VHA) in the United States. Research is fundamental to the VHA mission; further, it considers QI critical to achieving the goal of becoming a learning healthcare system. Thus, participating in research and QI is an organizational culture component of the physician’s role, despite competing demands VHA clinicians often experience. Those interested in recruiting clinicians as either research or QI participants can approach them directly (once all ethical and regulatory approvals are complete); nonmonetary incentives, such as those previously mentioned, are not uncommon. If more extensive time is needed, researchers and QI project leaders may negotiate protected time for clinicians with facility leadership; but direct financial compensation is not exchanged. This philosophy could serve as a model for other systems attempting to encourage clinician participation in research and QI as subjects. For example, a recent study of primary care team coordination tested a multifaceted intervention consisting of monthly feedback reports and debriefs [10]. As this required all members of the primary care team to be present for the debrief, the research team coordinated with facility and regional network leaders to allow teams one hour a month for the teams to debrief. Indeed, protected time was found to be a valuable tool not only for recruitment but also for exposure to the intervention, as teams who debriefed consistently exhibited better outcomes than teams who did not [11].

## Conclusion

Research and QI are integral to the betterment of medicine and healthcare. Results from the research and QI activities in which physicians participate can bring new approaches to medical practice that improve workflow efficiencies, reduce errors and improve patient satisfaction. This benefits the practice financially *and* serves the greater good of healthcare. Further, clinicians serving as research or QI subjects can personally benefit from a continuous learning perspective. To increase the supply of physician subjects, new models are needed to clarify the physician’s role in research and QI as a subject, and to successfully and ethically encourage and incorporate these activities in clinician agendas through non-monetary means. Explicit ethical guidance would constitute an important and beneficial, if insufficient first step.

## Data Availability

Not applicable.

## Abbreviations

QI: Quality improvement
AMA: American Medical Association
RVU: Relative value unit
VHA: Veterans Health Administration
